# Supplementary material for: Dynalign II: common secondary structure prediction for RNA homologs with domain insertions
Source: Nucleic Acids Res. 2014 Nov 21;42(22):13939–48. doi: 10.1093/nar/gku1172 (PMC4267632; doi:10.1093/nar/gku1172)
Supplement: SUPPLEMENTARY DATA [file supp_gku1172_nar-02021-z-2014-File012.zip › manual/javadoc/RNAstructure_java_interface/source/windows/FoldDoubleWindow.html]

FoldDoubleWindow


JavaScript is disabled on your browser.


- Overview
- Package
- Class
- Tree
- Index
- Help

- Prev Class
- Next Class

- Frames
- No Frames

- All Classes

- Summary:
- Nested |
- Field |
- Constr |
- Method

- Detail:
- Field |
- Constr |
- Method


RNAstructure\_java\_interface.source.windows

## Class FoldDoubleWindow

- java.lang.Object
- - java.awt.Component
  - - java.awt.Container
    - - javax.swing.JComponent
      - - javax.swing.JInternalFrame
        - - RNAstructure\_java\_interface.source.windows.InternalWindow
          - - RNAstructure\_java\_interface.source.windows.ModuleWindow
            - - RNAstructure\_java\_interface.source.windows.FoldDoubleWindow

- All Implemented Interfaces:
  :   ActionListener, ImageObserver, MenuContainer, Serializable, EventListener, Accessible, RootPaneContainer, WindowConstants

  ---

    

  ```
  public class FoldDoubleWindow
  extends ModuleWindow
  ```

  A class responsible for initializing and running the bifold module, which
  folds two strands of nucleic acids into their likely hybrid structure.

  See Also:
  :   Serialized Form

- - ### Nested Class Summary

    - ### Nested classes/interfaces inherited from class javax.swing.JInternalFrame

      `JInternalFrame.AccessibleJInternalFrame, JInternalFrame.JDesktopIcon`
    - ### Nested classes/interfaces inherited from class javax.swing.JComponent

      `JComponent.AccessibleJComponent`
    - ### Nested classes/interfaces inherited from class java.awt.Container

      `Container.AccessibleAWTContainer`
    - ### Nested classes/interfaces inherited from class java.awt.Component

      `Component.AccessibleAWTComponent, Component.BaselineResizeBehavior, Component.BltBufferStrategy, Component.FlipBufferStrategy`
  - ### Field Summary

    Fields

    | Modifier and Type | Field and Description |
    | --- | --- |
    | `private static long` | `serialVersionUID` |

    - ### Fields inherited from class RNAstructure\_java\_interface.source.windows.ModuleWindow

      `isRNA, progressBar`
    - ### Fields inherited from class RNAstructure\_java\_interface.source.windows.InternalWindow

      `backend, dialogHandler, menuBar`
    - ### Fields inherited from class javax.swing.JInternalFrame

      `closable, CONTENT_PANE_PROPERTY, desktopIcon, FRAME_ICON_PROPERTY, frameIcon, GLASS_PANE_PROPERTY, iconable, IS_CLOSED_PROPERTY, IS_ICON_PROPERTY, IS_MAXIMUM_PROPERTY, IS_SELECTED_PROPERTY, isClosed, isIcon, isMaximum, isSelected, LAYERED_PANE_PROPERTY, maximizable, MENU_BAR_PROPERTY, resizable, ROOT_PANE_PROPERTY, rootPane, rootPaneCheckingEnabled, title, TITLE_PROPERTY`
    - ### Fields inherited from class javax.swing.JComponent

      `accessibleContext, listenerList, TOOL_TIP_TEXT_KEY, ui, UNDEFINED_CONDITION, WHEN_ANCESTOR_OF_FOCUSED_COMPONENT, WHEN_FOCUSED, WHEN_IN_FOCUSED_WINDOW`
    - ### Fields inherited from class java.awt.Component

      `BOTTOM_ALIGNMENT, CENTER_ALIGNMENT, LEFT_ALIGNMENT, RIGHT_ALIGNMENT, TOP_ALIGNMENT`
    - ### Fields inherited from interface javax.swing.WindowConstants

      `DISPOSE_ON_CLOSE, DO_NOTHING_ON_CLOSE, EXIT_ON_CLOSE, HIDE_ON_CLOSE`
    - ### Fields inherited from interface java.awt.image.ImageObserver

      `ABORT, ALLBITS, ERROR, FRAMEBITS, HEIGHT, PROPERTIES, SOMEBITS, WIDTH`
  - ### Constructor Summary

    Constructors

    Constructor and Description

    `FoldDoubleWindow(String acid)`

    Constructor.
  - ### Method Summary

    Methods

    | Modifier and Type | Method and Description |
    | --- | --- |
    | `protected void` | `executeModuleAction(String command)` Execute an action within a particular module. |
    | `protected void` | `makeInputControls()` Make any input controls necessary to get data from this window for a calculation. |
    | `protected void` | `runMainCalculation()` Run the module calculation in the back end. |
    | `protected RolloverMenu[]` | `setMenus()` Create the array of variable menus used by this window. |

    - ### Methods inherited from class RNAstructure\_java\_interface.source.windows.ModuleWindow

      `combineFileNames, doActions, finishModule, getInputControl, getInputControl, makeStartButton, moduleInitialized, placeComponent, replaceExtension, setAnchorCenter, setAnchorNorth, setFillCenter, setFillHorizontal, setGrid, setInsets, setPad, showProgressBarDeterminate, showProgressBarIndeterminate`
    - ### Methods inherited from class RNAstructure\_java\_interface.source.windows.InternalWindow

      `actionPerformed, setTitles, viewWindow`
    - ### Methods inherited from class javax.swing.JInternalFrame

      `addImpl, addInternalFrameListener, createRootPane, dispose, doDefaultCloseAction, fireInternalFrameEvent, getAccessibleContext, getContentPane, getDefaultCloseOperation, getDesktopIcon, getDesktopPane, getFocusCycleRootAncestor, getFocusOwner, getFrameIcon, getGlassPane, getInternalFrameListeners, getJMenuBar, getLastCursor, getLayer, getLayeredPane, getMenuBar, getMostRecentFocusOwner, getNormalBounds, getRootPane, getTitle, getUI, getUIClassID, getWarningString, hide, isClosable, isClosed, isFocusCycleRoot, isIcon, isIconifiable, isMaximizable, isMaximum, isResizable, isRootPaneCheckingEnabled, isSelected, moveToBack, moveToFront, pack, paintComponent, paramString, remove, removeInternalFrameListener, reshape, restoreSubcomponentFocus, setClosable, setClosed, setContentPane, setCursor, setDefaultCloseOperation, setDesktopIcon, setFocusCycleRoot, setFrameIcon, setGlassPane, setIcon, setIconifiable, setJMenuBar, setLayer, setLayer, setLayeredPane, setLayout, setMaximizable, setMaximum, setMenuBar, setNormalBounds, setResizable, setRootPane, setRootPaneCheckingEnabled, setSelected, setTitle, setUI, show, toBack, toFront, updateUI`
    - ### Methods inherited from class javax.swing.JComponent

      `addAncestorListener, addNotify, addVetoableChangeListener, computeVisibleRect, contains, createToolTip, disable, enable, firePropertyChange, firePropertyChange, firePropertyChange, fireVetoableChange, getActionForKeyStroke, getActionMap, getAlignmentX, getAlignmentY, getAncestorListeners, getAutoscrolls, getBaseline, getBaselineResizeBehavior, getBorder, getBounds, getClientProperty, getComponentGraphics, getComponentPopupMenu, getConditionForKeyStroke, getDebugGraphicsOptions, getDefaultLocale, getFontMetrics, getGraphics, getHeight, getInheritsPopupMenu, getInputMap, getInputMap, getInputVerifier, getInsets, getInsets, getListeners, getLocation, getMaximumSize, getMinimumSize, getNextFocusableComponent, getPopupLocation, getPreferredSize, getRegisteredKeyStrokes, getSize, getToolTipLocation, getToolTipText, getToolTipText, getTopLevelAncestor, getTransferHandler, getVerifyInputWhenFocusTarget, getVetoableChangeListeners, getVisibleRect, getWidth, getX, getY, grabFocus, isDoubleBuffered, isLightweightComponent, isManagingFocus, isOpaque, isOptimizedDrawingEnabled, isPaintingForPrint, isPaintingOrigin, isPaintingTile, isRequestFocusEnabled, isValidateRoot, paint, paintBorder, paintChildren, paintImmediately, paintImmediately, print, printAll, printBorder, printChildren, printComponent, processComponentKeyEvent, processKeyBinding, processKeyEvent, processMouseEvent, processMouseMotionEvent, putClientProperty, registerKeyboardAction, registerKeyboardAction, removeAncestorListener, removeNotify, removeVetoableChangeListener, repaint, repaint, requestDefaultFocus, requestFocus, requestFocus, requestFocusInWindow, requestFocusInWindow, resetKeyboardActions, revalidate, scrollRectToVisible, setActionMap, setAlignmentX, setAlignmentY, setAutoscrolls, setBackground, setBorder, setComponentPopupMenu, setDebugGraphicsOptions, setDefaultLocale, setDoubleBuffered, setEnabled, setFocusTraversalKeys, setFont, setForeground, setInheritsPopupMenu, setInputMap, setInputVerifier, setMaximumSize, setMinimumSize, setNextFocusableComponent, setOpaque, setPreferredSize, setRequestFocusEnabled, setToolTipText, setTransferHandler, setUI, setVerifyInputWhenFocusTarget, setVisible, unregisterKeyboardAction, update`
    - ### Methods inherited from class java.awt.Container

      `add, add, add, add, add, addContainerListener, addPropertyChangeListener, addPropertyChangeListener, applyComponentOrientation, areFocusTraversalKeysSet, countComponents, deliverEvent, doLayout, findComponentAt, findComponentAt, getComponent, getComponentAt, getComponentAt, getComponentCount, getComponents, getComponentZOrder, getContainerListeners, getFocusTraversalKeys, getFocusTraversalPolicy, getLayout, getMousePosition, insets, invalidate, isAncestorOf, isFocusCycleRoot, isFocusTraversalPolicyProvider, isFocusTraversalPolicySet, layout, list, list, locate, minimumSize, paintComponents, preferredSize, printComponents, processContainerEvent, processEvent, remove, removeAll, removeContainerListener, setComponentZOrder, setFocusTraversalPolicy, setFocusTraversalPolicyProvider, transferFocusDownCycle, validate, validateTree`
    - ### Methods inherited from class java.awt.Component

      `action, add, addComponentListener, addFocusListener, addHierarchyBoundsListener, addHierarchyListener, addInputMethodListener, addKeyListener, addMouseListener, addMouseMotionListener, addMouseWheelListener, bounds, checkImage, checkImage, coalesceEvents, contains, createImage, createImage, createVolatileImage, createVolatileImage, disableEvents, dispatchEvent, enable, enableEvents, enableInputMethods, firePropertyChange, firePropertyChange, firePropertyChange, firePropertyChange, firePropertyChange, firePropertyChange, getBackground, getBounds, getColorModel, getComponentListeners, getComponentOrientation, getCursor, getDropTarget, getFocusListeners, getFocusTraversalKeysEnabled, getFont, getForeground, getGraphicsConfiguration, getHierarchyBoundsListeners, getHierarchyListeners, getIgnoreRepaint, getInputContext, getInputMethodListeners, getInputMethodRequests, getKeyListeners, getLocale, getLocation, getLocationOnScreen, getMouseListeners, getMouseMotionListeners, getMousePosition, getMouseWheelListeners, getName, getParent, getPeer, getPropertyChangeListeners, getPropertyChangeListeners, getSize, getToolkit, getTreeLock, gotFocus, handleEvent, hasFocus, imageUpdate, inside, isBackgroundSet, isCursorSet, isDisplayable, isEnabled, isFocusable, isFocusOwner, isFocusTraversable, isFontSet, isForegroundSet, isLightweight, isMaximumSizeSet, isMinimumSizeSet, isPreferredSizeSet, isShowing, isValid, isVisible, keyDown, keyUp, list, list, list, location, lostFocus, mouseDown, mouseDrag, mouseEnter, mouseExit, mouseMove, mouseUp, move, nextFocus, paintAll, postEvent, prepareImage, prepareImage, processComponentEvent, processFocusEvent, processHierarchyBoundsEvent, processHierarchyEvent, processInputMethodEvent, processMouseWheelEvent, remove, removeComponentListener, removeFocusListener, removeHierarchyBoundsListener, removeHierarchyListener, removeInputMethodListener, removeKeyListener, removeMouseListener, removeMouseMotionListener, removeMouseWheelListener, removePropertyChangeListener, removePropertyChangeListener, repaint, repaint, repaint, resize, resize, setBounds, setBounds, setComponentOrientation, setDropTarget, setFocusable, setFocusTraversalKeysEnabled, setIgnoreRepaint, setLocale, setLocation, setLocation, setName, setSize, setSize, show, size, toString, transferFocus, transferFocusBackward, transferFocusUpCycle`
    - ### Methods inherited from class java.lang.Object

      `clone, equals, finalize, getClass, hashCode, notify, notifyAll, wait, wait, wait`

- - ### Field Detail


    - #### serialVersionUID

      ```
      private static final long serialVersionUID
      ```

      See Also:
      :   Constant Field Values
  - ### Constructor Detail


    - #### FoldDoubleWindow

      ```
      public FoldDoubleWindow(String acid)
      ```

      Constructor.

      Parameters:
      :   `acid` - The nucleic acid type.
  - ### Method Detail


    - #### executeModuleAction

      ```
      protected void executeModuleAction(String command)
      ```

      **Description copied from class: `ModuleWindow`**

      Execute an action within a particular module.

      **Specified by:**
      :   `executeModuleAction` in class `ModuleWindow`

      Parameters:
      :   `command` - The command that signifies a particular action.


    - #### makeInputControls

      ```
      protected void makeInputControls()
      ```

      **Description copied from class: `ModuleWindow`**

      Make any input controls necessary to get data from this window for a
      calculation.

      **Specified by:**
      :   `makeInputControls` in class `ModuleWindow`


    - #### runMainCalculation

      ```
      protected void runMainCalculation()
      ```

      **Description copied from class: `ModuleWindow`**

      Run the module calculation in the back end.
        
        
      Data must be pulled from the module window before the back end
      calculation is run, so this data capture should be done in this method.

      **Specified by:**
      :   `runMainCalculation` in class `ModuleWindow`


    - #### setMenus

      ```
      protected RolloverMenu[] setMenus()
      ```

      **Description copied from class: `InternalWindow`**

      Create the array of variable menus used by this window.

      **Specified by:**
      :   `setMenus` in class `InternalWindow`

      Returns:
      :   The array of variable menus created for this window.


- Overview
- Package
- Class
- Tree
- Index
- Help

- Prev Class
- Next Class

- Frames
- No Frames

- All Classes

- Summary:
- Nested |
- Field |
- Constr |
- Method

- Detail:
- Field |
- Constr |
- Method
